# Case Report: Synchronous double primary male breast cancer and non-small cell lung cancer: diagnostic confirmation and multidisciplinary management

**DOI:** 10.3389/fmed.2026.1950636

**Published:** 2026-10-01

**Authors:** Ye Zhang, Lei Cao, Yu Zheng, Yang Yang, Xiaoqin Guan, Xiangxin Zheng, Peng Yang, Weiyou Zhu

**Affiliations:** 1 Department of Oncology, Jiangsu Province (Suqian) Hospital, Suqian, Jiangsu, China; 2 Department of Pathology, Jiangsu Province (Suqian) Hospital, Suqian, Jiangsu, China; 3 Department of Thyroid and Breast Surgery, Jiangsu Province (Suqian) Hospital, Suqian, Jiangsu, China; 4 Department of Oncology, The First Affiliated Hospital of Nanjing Medical University, Nanjing, Jiangsu, China

**Keywords:** case report, concurrent thoracic radiotherapy, double primary cancer, male breast cancer, multidisciplinary team, non-small cell lung cancer, treatment prioritization

## Abstract

**Background:**

Male breast cancer (MBC) is rare, accounting for approximately 1% of all breast cancers. When MBC is diagnosed simultaneously with a second primary lung cancer, treatment planning becomes particularly complex because diagnostic certainty, technical resectability, competing treatment windows, and radiotherapy safety must all be considered.

**Case presentation:**

We report a 72-year-old man who was synchronously diagnosed with left invasive ductal breast carcinoma and right lower lobe poorly differentiated non-small cell lung cancer (NSCLC). The breast cancer was staged as pT_3_N_2a_M_0_, AJCC 8th edition stage IIIA, with strong ER/PR expression and HER2 nonamplification. The lung lesion was pathologically diagnosed as poorly differentiated NSCLC without definitive glandular, squamous, or neuroendocrine differentiation and was staged as cT_3_N_0_M_0_, AJCC 8th edition stage IIB. Although stage IIB NSCLC is usually evaluated for surgery, this tumor closely involved the right lower lobe pulmonary vessels and bronchial structures, and thoracic surgeons considered upfront radical resection technically unsafe because of the risks of hemorrhage, extended resection, impaired blood supply to the remaining lobes, and loss of pulmonary function. After multidisciplinary team (MDT) discussion, the patient underwent left modified radical mastectomy, adjuvant AC-T chemotherapy, and simultaneous postmastectomy radiotherapy to the left chest wall/regional nodes with definitive radiotherapy to the right lung lesion plus concurrent cisplatin. Imaging shortly after radiotherapy showed marked shrinkage of the right lung lesion, but subsequent CT and liver MRI suggested systemic progression with multiple hepatic lesions. The patient declined liver biopsy, received one cycle of vinorelbine plus cisplatin with Endostar, discontinued further treatment, and died of liver failure in March 2026.

**Conclusion:**

This case illustrates that therapeutic prioritization in synchronous double primary cancers should not be determined solely by TNM stage or chronological order of diagnosis. Instead, treatment decisions should integrate pathological distinction, tumor biology, anatomical treatability, technical resectability, curative potential, competing treatment windows, and short-term clinical threat. In selected patients, simultaneous postmastectomy radiotherapy and definitive lung radiotherapy may be feasible when supported by MDT consensus, dosimetric review, and close toxicity monitoring.

## Introduction

Breast cancer and lung cancer are among the most common malignancies worldwide, whereas male breast cancer is rare and accounts for less than 1% of all breast cancers (1–7). Because large prospective datasets for MBC are limited, most therapeutic principles are extrapolated from female breast cancer (8). Non-small cell lung cancer accounts for the majority of lung cancers and includes a broad spectrum of histological and molecular subtypes (9, 10).

Multiple primary malignant tumors are defined as independent malignant tumors arising in the same patient, rather than recurrence or metastasis from each other (11–16). According to classical Warren and Gates criteria, each tumor should be malignant, should have distinct pathological features, and should not represent metastasis from another tumor (11). For breast cancer patients who present with a new pulmonary lesion, the key clinical question is whether the lesion represents lung metastasis or a second primary lung cancer, as the treatment pathways differ fundamentally.

Even after the diagnosis of synchronous double primary cancers is established, the treatment sequence may remain challenging. Decisions must account not only for TNM stage but also for tumor biology, anatomical treatability, technical resectability, organ preservation, expected natural history, and toxicity from overlapping treatments. We report a rare case of synchronous MBC and technically unresectable stage IIB poorly differentiated NSCLC, focusing on three practical questions: how the diagnosis of double primary cancers was supported, why the stage IIB lung cancer was not treated surgically, and why postmastectomy radiotherapy and definitive lung radiotherapy were delivered during the same treatment period.

## Case presentation

### Initial presentation and diagnosis of synchronous double primary malignancies

A 72-year-old man presented on March 27, 2025, with a one-year history of a left breast mass. Superficial ultrasound on April 28, 2025, revealed a left breast nodule classified as BI-RADS 4c. A preoperative chest CT performed on the same day incidentally showed a 56 mm × 27 mm mass in the dorsal segment of the right lower lobe, highly suspicious for primary lung cancer.

Because of the suspicious pulmonary mass, CT-guided lung biopsy was performed on April 30, 2025. Pathology showed poorly differentiated non-small cell carcinoma without definite glandular, squamous, or neuroendocrine differentiation. Immunohistochemistry showed Napsin A (-), mammaglobin (-), ER (-), PR (-), HER2 (-), p40 (-), Syn (-), CgA (-), focal GATA3 positivity, Ki67 of approximately 70% in hotspot areas, p53 overexpression (approximately 80%, 2+, suggestive of an aberrant pattern), weak BRG1 expression, and CK-L positivity (Figure 1). PD-L1 testing showed TPS <1% and CPS 7. No clinically actionable alterations were detected in EGFR, ALK, ROS1, MET, or BRAF, and the tumor was microsatellite stable. Taken together, the morphology, immunophenotype, molecular profile, and radiological presentation supported primary poorly differentiated NSCLC.

**Figure 1:**
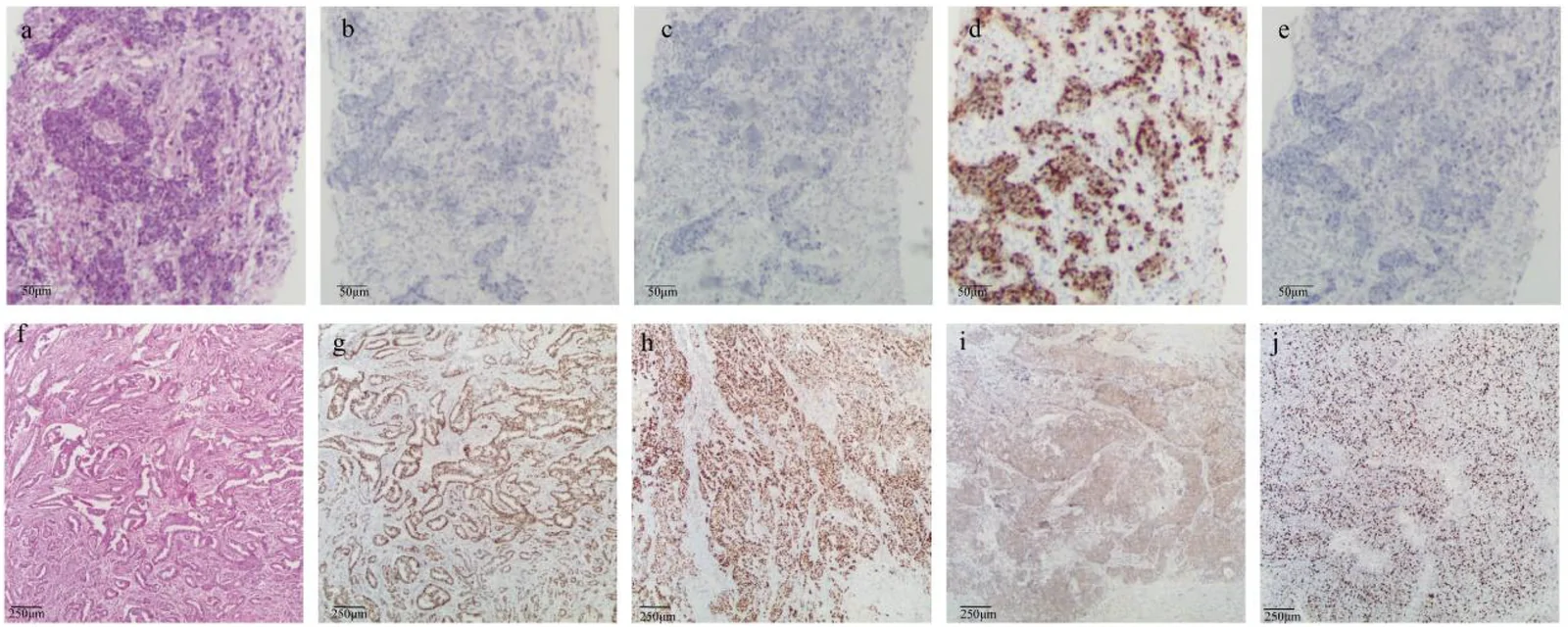
Representative pathological findings of the lung and breast tumors. Panels **a–e** show hematoxylin and eosin staining and immunohistochemical expression of p40, Napsin A, Ki67, and CgA in the lung tumor. Panels **f–j** show hematoxylin and eosin staining and immunohistochemical expression of ER, PR, HER2, and Ki67 in the breast tumor.

Core needle biopsy of the left breast mass on May 5, 2025, revealed invasive ductal carcinoma. Fine needle aspiration of the left axillary lymph node showed malignant tumor cells consistent with nodal metastasis. A left modified radical mastectomy was performed on May 6, 2025. The standard procedure included a complete axillary lymph node dissection (ALND). Intraoperatively, sentinel lymph node biopsy was performed first (6 nodes were identified, of which 3 were positive), followed by ipsilateral axillary levels I and II lymph node dissections. A total of 17 lymph nodes were submitted for pathological examination, of which 3 were positive for metastasis. Postoperative pathology showed invasive ductal carcinoma, grade III, measuring 5.3 cm × 5.0 cm × 3.5 cm. Immunohistochemistry showed ER >95% (2 + to 3+), PR >90% in hotspot areas (2 + to 3+), HER2 2+, Ki67 30%–40%, and CK5/6 (-). FISH showed no HER2 amplification. The breast cancer was staged as pT_3_N_2a_M_0_, AJCC 8th edition stage IIIA, and classified as Luminal B, HER2-negative. The lung cancer was staged as cT_3_N_0_M_0_, AJCC 8th edition stage IIB. The overall findings supported synchronous double primary malignancies involving the left breast and right lung.

### MDT assessment and treatment prioritization

The first major treatment question was why the stage IIB lung cancer was not surgically treated. Although the right lower lobe tumor was staged as cT_3_N_0_M_0_, imaging demonstrated close involvement of the right lower lobe pulmonary vessels and bronchial structures. The surgical planes were considered unclear, and thoracic surgeons judged that forced dissection could lead to major intraoperative hemorrhage. If extended resection were performed to ensure margin clearance and surgical safety, the blood supply to the right upper and middle lobes might be compromised, with substantial loss of postoperative pulmonary function and increased perioperative risk. Therefore, the decision was not based solely on TNM stage. Instead, the MDT integrated anatomical location, vascular and airway involvement, technical resectability, lung function preservation, and perioperative risk and concluded that upfront radical pulmonary resection was not appropriate.

Because the breast cancer had a clear surgical indication and could be locally controlled by modified radical mastectomy, surgery for the left breast cancer was performed first. The lung cancer, while not surgically resectable, still had a potential curative treatment window with definitive radiotherapy and concurrent platinum-based chemotherapy. The treatment plan was therefore designed to avoid prolonged delays in lung-directed definitive therapy while preserving standard adjuvant treatment for high-risk breast cancer.

### Treatment course

After a left modified radical mastectomy, the patient received adjuvant AC-T chemotherapy beginning on May 28, 2025, consisting of four cycles of anthracycline plus cyclophosphamide followed by four cycles of taxane. The last cycle was completed on November 3, 2025. During adjuvant chemotherapy for breast cancer, the right lung lesion showed only transient and inconsistent changes: it was slightly reduced after two cycles of chemotherapy and assessed as stable disease, but it enlarged after completion of eight cycles and was assessed as progressive disease. This observation suggested that breast cancer-directed systemic therapy could not provide reliable and durable control of the lung tumor.

A second MDT meeting supported simultaneous radiotherapy rather than sequential treatment. The rationale was based on seven considerations. First, the lung cancer was technically unsuitable for surgery and required prompt definitive local treatment. Second, the breast cancer was pT3N2aM0, stage IIIA, with nodal involvement and had a clear indication for postmastectomy chest wall and regional nodal irradiation. Third, sequential radiotherapy would have further prolonged the interval before lung-directed definitive treatment. Fourth, the AC-T regimen had not achieved durable control of the lung lesion. Fifth, the left chest wall/regional nodal target and the right lower lobe lung target were anatomically separated, allowing a treatment plan that evaluated cumulative doses to the lungs, heart, esophagus, and spinal cord. Sixth, concurrent scheduling shortened the total treatment duration and improved the possibility of completing comprehensive treatment for both malignancies. Seventh, the expected acute toxicity was considered manageable under close monitoring.

From November 27, 2025, the patient received concurrent radiotherapy to the left chest wall/regional nodes and the right lung lesion. The treatment was delivered using fixed-field intensity-modulated radiotherapy (IMRT). A single set of planning CT images was used to define both the breast/chest wall targets and the lung target. Separate isocenters were placed according to the anatomical location of each target volume. Two independent IMRT plans were generated and the doses were vector-summed to calculate and evaluate the cumulative dose to each organ at risk. This approach was selected because it allowed adequate coverage of both the left chest wall/regional nodal areas and the right lower lobe lesion while effectively sparing adjacent organs at risk. The postmastectomy radiotherapy to the left chest wall and left supraclavicular/infraclavicular lymphatic drainage regions was delivered using 6 MV x-rays, with a prescription dose of 50 Gy in 25 fractions, 2 Gy per fraction, 5 fractions per week, with a bolus during the first 15 fractions. During the same treatment period, definitive radiotherapy was delivered to the right lung lesion with PTV-lung covering the pulmonary tumor at 60 Gy in 30 fractions, 2 Gy per fraction, 5 fractions per week, combined with concurrent weekly cisplatin 50 mg. The available plan summary is shown in Table 1. Major acute adverse events included grade III leukopenia, grade I nausea, and decreased appetite. No clinically apparent radiation pneumonitis or esophagitis was observed during the recorded treatment period.

**Table 1:** Dosimetric parameters of simultaneous postmastectomy and right lung radiotherapy.

Organ at risk	Parameter	Value
Heart	Dmean	10.76 Gy
Heart	V5	60%
Left lung	Dmean	14.20 Gy
Left lung	V20	25.68%
Left lung	V5	55%
Right lung	V5	52.9%
Right lung	V30	8.9%
Spinal cord PRV	Dmax	41.13 Gy
Left glenohumeral joint	V30	21.1%
Thyroid	Dmean	25.64 Gy
Esophagus	V20	23%
Liver	V5	6.68%
Carotid artery	V40	0%

Dmean, mean dose; Dmax, maximum dose; Vx, percentage volume receiving at least x Gy; PRV, planning organ-at-risk volume.

### Response assessment and follow-up

Serial CT imaging showed that the right lung lesion decreased markedly after lung-directed radiotherapy and was assessed as a partial response. However, on February 2, 2026, tumor markers rose substantially compared with previous normal levels (Figure 2A). A chest CT on February 2, 2026, and a liver-enhanced MRI on February 5, 2026, showed that the right lower lobe primary lesion was approximately stable, while multiple bilateral pulmonary nodules were slightly enlarged, mediastinal lymph nodes were enlarged, and multiple abnormal enhancing hepatic lesions were radiologically suspicious for metastases. T12 and L1 vertebral changes were also noted (Figure 3).

**Figure 2:**
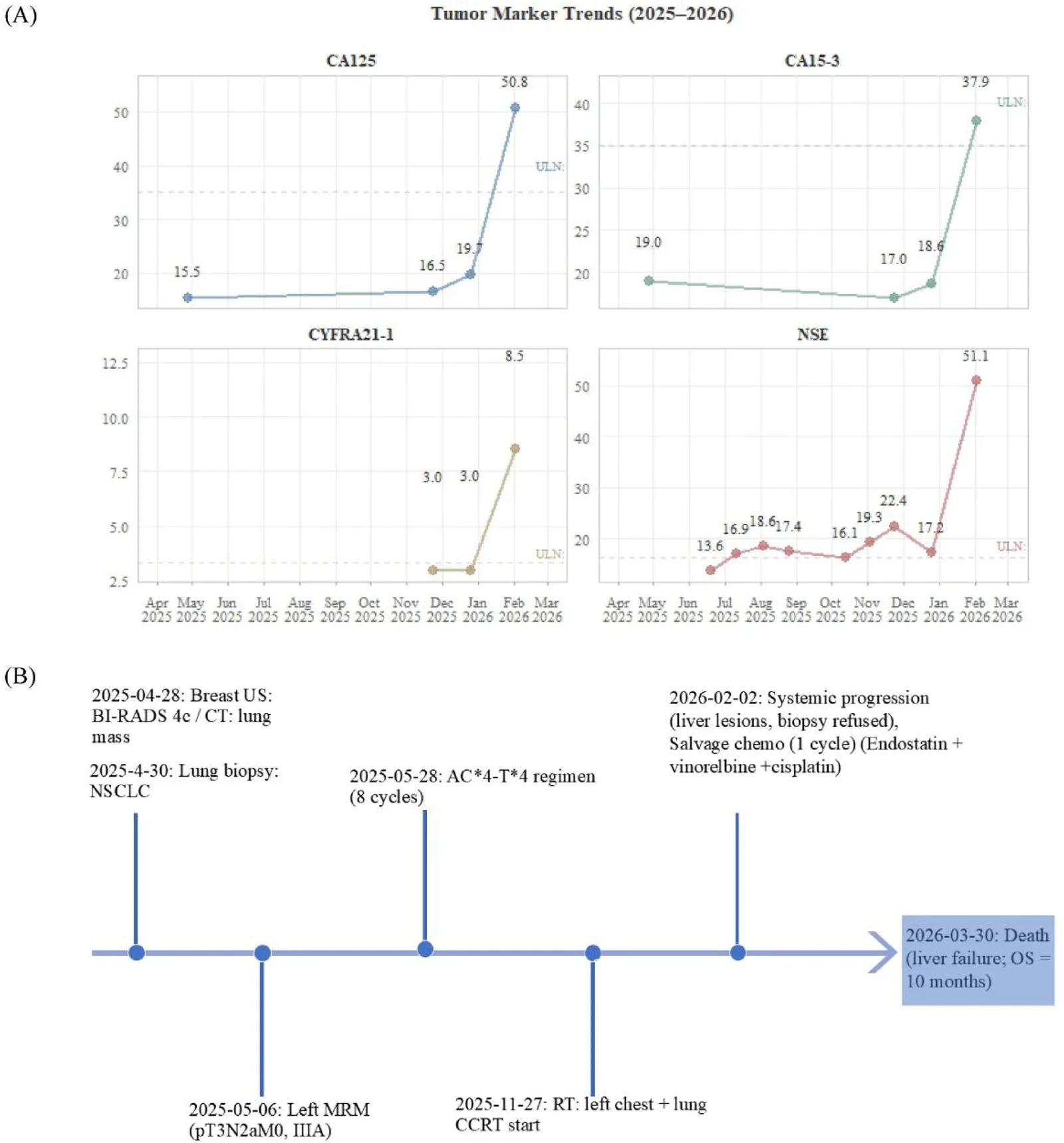
Tumor marker trends and clinical timeline. Panel **A** shows changes in CA125, CA15-3, CYFRA21-1, and NSE during the disease course. Panel **B** summarizes the main diagnostic, therapeutic, progression, and outcome events.

**Figure 3:**
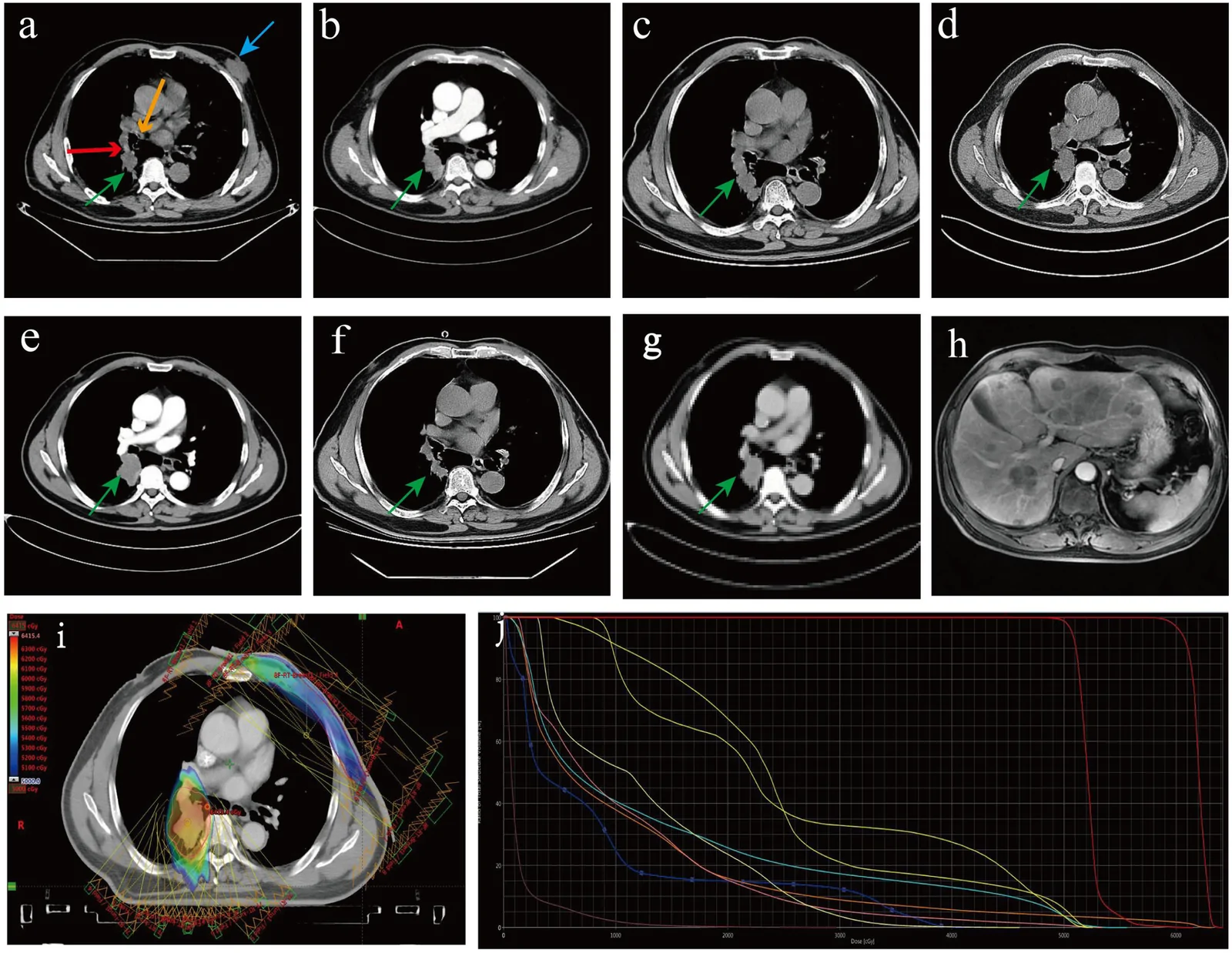
Serial imaging and radiotherapy planning during the treatment course. **(a)** Baseline chest CT (April 28, 2025) showing the right lower lobe mass with close involvement of pulmonary vessels and bronchial structures. The red arrow indicates the first-level branch of the pulmonary artery in the lower lobe of the right lung encased by the tumor; The yellow arrow indicates the posterior segment bronchus; the blue arrow points to the breast lesion; the green arrow points to the lung lesion. **(b–f)** Serial CT changes during chemotherapy and radiotherapy: **(b)** before the first cycle of chemotherapy (May 30, 2025); **(c)** after two cycles of chemotherapy (July 21, 2025); **(d)** after completion of eight cycles of chemotherapy (November 3, 2025); **(e)** baseline assessment before radiotherapy (November 25, 2025); **(f)** post-radiotherapy reassessment (December 30, 2025). The green arrow points to the lung lesion. **(g)** Final follow-up CT scan (February 2, 2026). **(h)** Contrast-enhanced liver MRI (February 5, 2026) showing multiple hepatic lesions radiologically suspicious for metastases. **(i,j)** Radiotherapy field arrangement and dose distribution. DVH: Yellow: thyroid; dark blue: spinal cord; orange: right lung; light blue: left lung; brown: liver; pink: heart; pale yellow: esophagus).

The findings were considered radiologically suspected systemic progression, but the tissue origin of the liver lesions was not pathologically confirmed. Following the completion of adjuvant radiotherapy, the patient was started on daily oral tamoxifen as adjuvant endocrine therapy for hormone receptor-positive breast cancer. However, after approximately two weeks of tamoxifen administration, a restaging CT scan revealed disease progression. Tamoxifen was therefore discontinued, and systemic therapy was initiated. Liver biopsy was recommended to clarify whether the systemic progression originated from breast cancer, lung cancer, or both; however, the patient and his family declined biopsy. As the origin of the liver lesions was unknown, we chose vinorelbine/cisplatin/Endostar to target both possible primary tumors. This regimen is standard for NSCLC, and Endostar offers additional broad-spectrum activity—a pragmatic choice without pathological confirmation. On February 5, 2026, the patient received one cycle of Endostar (30 mg continuous intravenous infusion over 24 h, days 1–7), vinorelbine soft capsule 100 mg on days 1 and 8, and cisplatin 70 mg on days 1 and 2, every three weeks. The patient subsequently discontinued treatment and died of liver failure in March 2026, with an overall survival of approximately 10 months from initial diagnosis (Figure 2B).

## Discussion

### Confirmation of synchronous double primary malignancies

Based on the pathological and immunohistochemical findings, the diagnosis of primary breast cancer and non-small cell lung cancer was confirmed. The first question in this case was how to establish the diagnosis of double primary cancers. A pulmonary mass in a patient with newly diagnosed breast cancer can easily be interpreted as metastatic disease, and prior series have shown that synchronous breast and primary lung cancers require careful multidisciplinary assessment (17–19). In this patient, however, the two tumors showed distinct pathological and immunophenotypic profiles. The breast tumor was invasive ductal carcinoma with strong ER and PR expression and no HER2 amplification, whereas the lung lesion was poorly differentiated non-small cell carcinoma with negative ER, PR, mammaglobin, HER2, p40, Napsin A, Syn, and CgA and a higher proliferative index. Together with the presence of a dominant right lower lobe mass and the absence of clinicopathological evidence supporting breast cancer metastasis as the sole explanation, these findings support synchronous double primary malignancies rather than metastatic breast cancer to the lung.

The diagnosis had direct therapeutic implications. If the lung lesion had been interpreted as breast cancer metastasis, management would have shifted toward systemic therapy for stage IV breast cancer. In contrast, a second primary localized NSCLC required an assessment of local radical treatment options and technical resectability. The value of this case, therefore, lies not simply in its rarity but in the need to distinguish tumor origin and then prioritize treatment under competing oncological timelines.

### Why stage IIB lung cancer was not treated surgically

The second question was why a cT_3_N_0_M_0_ stage IIB lung cancer was not managed with upfront surgery. This case illustrates that TNM stage alone is insufficient to determine operability. Guidelines for early and locally advanced NSCLC generally support surgical evaluation for resectable stage II disease, but they also emphasize individualized decision-making when tumor location, cardiopulmonary reserve, and technical resectability are limiting factors (20–22). Although the absence of nodal disease would usually prompt surgical consideration, the tumor was anatomically complex, with close involvement of the lower lobe pulmonary vessels and bronchial structures. Imaging revealed that the lesion was located in the dorsal segment of the right lower lobe, where it encased the first-order branches of the right lower lobe pulmonary artery and the dorsal segmental bronchus. Thoracic surgeons considered that forced resection could lead to major intraoperative hemorrhage. Moreover, if extensive right lower lobe resection were performed to ensure margin clearance and surgical safety, the blood supply to the right upper and middle lobes might be compromised, resulting in clinically significant loss of postoperative pulmonary function and increased perioperative risk.

Therefore, the tumor was considered technically unsuitable for direct radical resection despite its stage IIB classification. The MDT decision emphasized technical resectability and functional preservation rather than stage alone. This distinction is important for case reporting: the lung cancer was not treated as an unresectable stage III tumor but as a localized stage IIB tumor whose anatomical relationship to vascular and airway structures made upfront surgery unsafe.

### Why upfront lung chemoradiotherapy was not prioritized before mastectomy

Although the lung cancer carried a worse overall prognosis, treatment sequencing in this patient was not determined solely by histological prognosis but by comprehensive MDT assessment weighing oncological urgency, therapeutic benefit, and normal tissue safety. The breast cancer was stage IIIA (pT3N2aM0) with axillary lymph node involvement, conferring a high risk of locoregional recurrence and a clear indication for timely mastectomy. Delaying mastectomy until after the completion of a full course of lung chemoradiotherapy would not have conferred any therapeutic benefit to the lung cancer, because the lung lesion had already been deemed technically unresectable and its curative treatment was radiotherapy-based rather than surgical. Such a delay, however, could have potentially increased the risk of local progression of breast cancer, as cautioned in breast cancer treatment guidelines. More importantly, if the patient had undergone upfront lung chemoradiotherapy followed by mastectomy and subsequent postmastectomy radiotherapy—which was indicated given the pT3N2aM0 status—the cervical and thoracic spinal cords would have been exposed to cumulative radiation doses from two independent treatment plans delivered within a short time frame, exceeding the safe tolerance threshold and elevating the risk of radiation myelopathy. By contrast, the chosen strategy—mastectomy followed by adjuvant AC-T chemotherapy and then concurrent radiotherapy to both the left chest wall/regional nodes and the right lung lesion—achieved timely local control of the breast cancer, avoided delay in lung-directed definitive therapy beyond the conventional window, and allowed for integrated radiation planning to constrain the cumulative spinal cord dose within a safe limit through unified plan optimization. This sequencing was therefore endorsed by the MDT as the most balanced approach for this patient.

### Why postmastectomy radiotherapy and definitive lung radiotherapy were delivered concurrently

The third and most distinctive management issue was the decision to deliver left-sided postmastectomy radiotherapy and right lower lobe definitive lung radiotherapy during the same treatment period. This concurrent approach was not chosen for convenience; it was an individualized MDT-based strategy. The lung cancer was technically unsuitable for surgery and required prompt definitive local treatment. The breast cancer, staged as pT_3_N_2a_M_0_ stage IIIA with nodal involvement, also had a clear indication for postmastectomy radiotherapy to the chest wall and regional lymphatic drainage areas (23–28). Definitive thoracic radiotherapy with appropriate systemic therapy is an accepted curative-intent strategy for selected medically or technically inoperable localized NSCLC, and evidence from lung cancer radiotherapy trials and guidelines supports careful integration of radiation dose, concurrent systemic therapy, and normal-tissue constraints (29–32). If the two radiotherapy courses had been delivered sequentially, lung-directed definitive therapy would have been delayed further, potentially increasing the risk of local progression or distant dissemination.

The dynamic response of the lung lesion during AC-T chemotherapy also influenced this decision. The lung lesion showed slight shrinkage after the early AC phase but enlarged after completion of the full AC-T regimen, suggesting that breast cancer-directed chemotherapy did not provide reliable durable control of the lung tumor. Therefore, it was inappropriate to rely on the breast adjuvant systemic regimen as sufficient therapy for lung cancer.

Technically, the left chest wall/regional nodal target and the right lower lobe lung target were spatially separated, allowing treatment planning to evaluate dose distribution across the lungs, heart, esophagus, and spinal cord. The available dosimetric data supported the feasibility of concurrent treatment. In this selected patient, the acute toxicity profile was acceptable and manageable. However, this observation is derived from a single case with limited follow-up; longer follow-up and larger series are required to assess late toxicity, including radiation-induced cardiotoxicity and pulmonary fibrosis. This concurrent strategy should therefore be reserved for carefully selected patients after MDT evaluation, rigorous dosimetric planning, and close toxicity monitoring, rather than generalized as a universally safe approach.

### Systemic progression, re-biopsy, and limitations

Although the right lung primary lesion showed a partial local response after radiotherapy, the patient rapidly developed radiologically suspected systemic progression with multiple liver lesions and rising tumor markers. The absence of pathological confirmation of the liver lesions represents a major limitation. Both breast cancer and poorly differentiated NSCLC can metastasize to the liver, and without biopsy or paired molecular comparison, it is not possible to definitively attribute the systemic progression or fatal liver failure to either primary tumor. This uncertainty limited the ability to select subsequent individualized therapy.

The case highlights a practical problem in double primary malignancies: when progression occurs, re-biopsy of metastatic lesions is particularly important because the origin of the progression may determine systemic treatment. Early communication with patients and families regarding the clinical value of re-biopsy should be part of the management plan. In this case, the patient declined liver biopsy and later discontinued therapy, reflecting the real-world challenges of treating aggressive double primary malignancies with limited tolerance and rapidly declining clinical status.

## Conclusion

This case of synchronous double primary malignancies in a 72-year-old man offers three lessons. First, double primary cancers should be confirmed by pathological and immunophenotypic assessment, not presumed to be metastasis. Second, TNM stage alone does not determine surgical resectability; vascular/bronchial involvement, technical feasibility, and functional preservation must be integrated. Third, simultaneous postmastectomy and definitive lung radiotherapy may be considered in selected patients when both tumors require timely treatment, targets are separated, dosimetric constraints are met, and toxicity is monitored. Biopsy of metastatic lesions at progression remains critical, especially in double primary cancers.

## Data Availability

The original contributions presented in the study are included in the article/Supplementary Material, further inquiries can be directed to the corresponding author.
